# Umbelliprenin Induces Apoptosis in CLL Cell Lines

**Published:** 2012

**Authors:** Seyed Ali Ziai, Omid Gholami, Mehrdad Iranshahi, Amir Hassan Zamani, Mahmood Jeddi-Tehrani

**Affiliations:** a *Pharmacology Department, Faculty of Medicine, Shahid Beheshti University of Medical Sciences, Tehran, Iran.*; b *Biotechnology Research Center and School of Pharmacy, Mashhad University of Medical Sciences, Mashhad, Iran. *; c *Nanobiotechnology Research Center, Avicenna Research Institute, ACECR, Tehran, Iran. *; d *Monoclonal Antibody Research Center, Avicenna Research Institute, ACECR, Tehran, Iran. *

**Keywords:** Chronic lymphocytic leukemia (CLL), Umbelliprenin, Apoptosis, Interleukin 4 (IL-4), Flowcytometry

## Abstract

Chronic lymphocytic leukemia (CLL) remains an incurable disease that requires innovative new approaches to improve therapeutic outcome. Many Ferula species, including *F. asa-foetida*, synthesize terpenyloxy coumarins. One of these coumarins is umbelliprenin, which has been implicated with induction of apoptosis in some cancer cell lines.

In this study induction of apoptosis by umbelliprenin on Jurkat T-CLL and Raji B-CLL cell lines was studied. In this regard, cells were incubated with various concentrations of umbelliprenin *in-vitro* for different times and assayed for apoptosis with annexin V–FITC/PI double staining flowcytometry method. Results showed that umbelliprenin induced apoptosis in leukemic cells in a dose- and time-dependent manner and that CLL cells were more susceptible to umbelliprenin induced cell death than normal peripheral blood mononuclear cell (PBMCs).

Moreover, we study the induction of apoptosis in Jurkat cells by umbelliprenin in the presence of interleukin 4 (IL-4) as an agent that causes resistance to apoptosis in CLL cells, was also student. We showed that IL-4 can not reduce apoptotic effect of umbelliprenin.

The preferential toxicity of umbelliprenin for CLL cells, supports the hypothesis that oral administration of umbelliprenin in the form of foods or folk medicines containing this coumarin, might enhance protection against the development of CLL in man with little side effects. In conclusion, umbelliprenin may be an effective therapeutic agent in the treatment of CLL, and thus clinical studies with umbelliprenin may be appropriate.

## Introduction

Chronic lymphocytic leukemia (CLL), a lymphoprolifrative disorder (lymphoid neoplasm), is usually described as the most common leukemia in the United States, Canada, and Western Europe, whereas it is rare in Japan and infrequent in other Asian societies (1). B-CLL is characterized by a progressive accumulation of functionally incompetent lymphocytes, which are monoclonal in origin (2).

**Figure 1:**
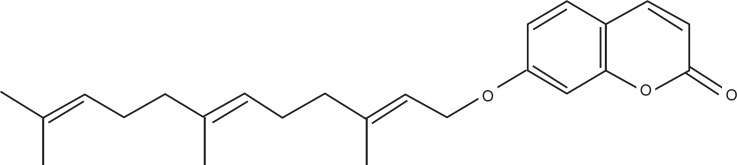
Structure of umbelliprenin.

Different species of the genus Ferula (Apiaceae) which is endemic in central Asia, are used as food and/or traditional medicine in Iran. The species *Ferula. asa-foetida* has been reported to exhibit anticarcinogenic properties and to afford protection against free radical- mediated diseases (3). Many Ferula species, including *Ferula. asa-foetida*, synthesize terpenyloxy coumarins. One of these coumarins has been named umbelliprenin (Figure1). It has been reported that umbelliprenin inhibits the red pigment production in *Serratia marcescens* (4), decreases matrix metalloprotease (MMP) activity (5) and exhibits antileishmanial activity against promastigotes (6).

Induction of apoptosis by umbelliprenin was first demonstrated in 2007. Barthomeuf *et al.* showed that umbelliprenin induces apoptosis in M4Beu malignant melanoma cell line (7). They have demonstrated that M4Beu cells are not only more susceptible to umbelliprenin compared with cisplatin, but are also markedly more sensitive to this coumarin than diploid fibroblasts (7). This point is important because it suggests a therapeutic margin.

Interleukin 4 (IL-4) is a cytokine known to be important in promoting the survival of CLL cells *in-vitro* at concentrations between 1 and 25 ng/mL as well as *in-vivo,* and may be an important factor in resistance to therapy (8-11). Also, the combination of IL-4 and fludarabine, a nucleoside analog used in the clinical management of B-CLL, causes most B-CLL cells to become less susceptible to fludarabine induced apoptosis (12).

 Considering these characteristics, the present work was designed to study the induction of apoptosis by umbelliprenin in Jurkat T-CLL and Raji B-CLL cell lines. Results showed that umbelliprenin could induce apoptosis in a dose and time dependent manner and that it kept its activity in the presence of the prosurvival cytokine interleukin-4 (IL-4).

## Experimental

*Plant material and umbelliprenin isolation*

Umbelliprenin (C24H30O3, MW: 366) was purified (> 95%) as previously described (8) from dried roots of *Ferula szowitsiana* D.C collected from the mountains of Golestan forest (Golestan province, Iran). A voucher specimen of the roots (no. M1001) has been deposited at the Department of Pharmacognosy and Biotechnology, Faculty of Pharmacy, Mashhad University of Medical Sciences, Iran.

*Cell culture*

CLL cell lines were prepared from National Cell Bank of Iran (Pasteur institute, Tehran, Iran). Cells were grown in RPMI 1640 culture medium containing 10% fetal bovine serum (FBS), Penicillin (10,000 U/mL) and Streptomycin (10 mg/mL) in several culture flasks in a CO_2_ (5%) incubator at 37°C and 95% humidity, until reaching a total cell count of 50×10^6^. Cells were then frozen in FBS containing 10% dimethyl sulfoxide (DMSO) and stored in liquid nitrogen (5×10^6^ cells/vial). The viability of cryopreserved cells was determined by trypan blue staining, immediately upon thawing. Only cells whose viability exceeded 93% (range of 93.4%-99%) were used in this study. Normal peripheral blood mononuclear cells (PBMC) were obtained from healthy volunteers, following an informed consent. PBMCs were isolated by Ficoll density-gradient centrifugation. Normal and cancerous cells were cultured in RPMI 1640 medium containing 10% FBS. 

*Apoptosis assay*

For apoptosis assay by flowcytometry, umbelliprenin was diluted in DMSO. Immediately before use, it was diluted in the culture medium to obtain a final DMSO concentration of 0.5% (v/v). Cells were cultured in a 24 well plate at a density of 10^6^ cells/well in RPMI 1640. Ten, 25, 50 and 100 µM concentrations of umbelliprenin were tested in triplicates at different times. Staurosporine was used as a positive control. Cells were removed from the culture plates, washed twice with cold PBS 1X, incubated in the dark at room temperature with annexin V–fluorescein isothiocyanate (FITC) and propidium iodide (PI; BD Bioscience, Material No. 556570) in 10× Annexin V binding buffer (diluted to 1×) for 15 min and evaluated for apoptosis by double staining flowcytometry (Partec Flomax) using FL1 and FL3 channels. To avoid nonspecific fluorescence from dead cells, live cells were gated tightly using forward and side scatter. For flowcytometric analysis of the experiments, negative control cells (cells incubated with medium alone) were stained with Annexin V-FITC and PI and gated according to the forward and side scatter. The parameters for this sample were saved and used to analyze apoptosis induction in the cells that were treated with umbelliprenin and staurosporine. Cells that were considered viable include are Annexin V-FITC and PI negative. Early apoptotic cells were Annexin V-FITC positive (indicating translocation of phosphatidylserine from the inner to the outer leaflet of the plasma membrane) and PI negative (with intact cellular membrane). Cells that were in late apoptosis or already dead include both the Annexin V-FITC and PI positive. 

*Induction of apoptosis by umbelliprenin in the presence of IL-4*

Jurkat T-CLL cells were cultured in a 24 well plate at a density of 10^6^ cells/well in RPMI 1640 containing 10% FBS and incubated with 1 ng/mL human IL-4 (BD, Biosciences, Cat. No. 354068). After 24 h, 50 µM umbelliprenin was added to the culture. After an additional 48 h, apoptosis was assayed by annexin V- FITC/PI double staining flowcytometry method as described above. 

*Statistical analysis*

One way ANOVA test was used for statistical analysis. The p-value was considered significant when it was less than 0.05.

**Figure 2:**
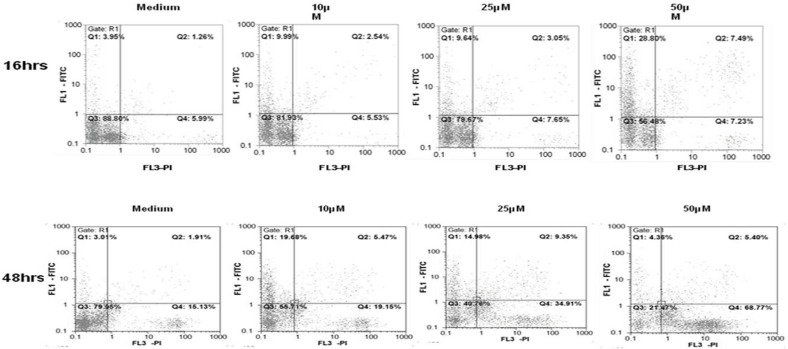
Annexin V-FITC/PI double staining flowcyotmetry analysis of apoptosis induction in Jurkat T-CLL cells by umbelliprenin (10, 25, 50 µM) after 16 and 48 h incubation. Numbers in each quadrant indicate the percentage of cells labeled with annexin V–FITC (top left), PI (bottom right), annexin V–FITC and PI (top right), or unlabeled (bottom left).

**Figure 3:**
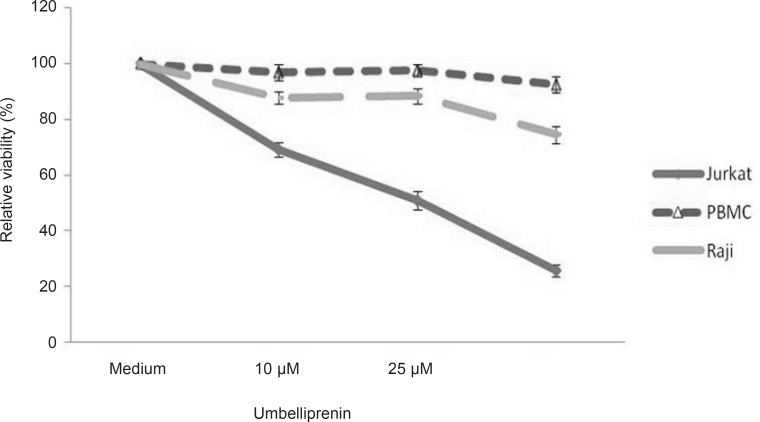
Relative percent viability of cells (Jurkat, Raji, PBMC) in the presence of umbelliprenin after 48 h incubation. Data is shown as mean ± standard deviation. Error bars represent 95% confidence interval. Viable cells are defined as those that were not stained neither with Annexin V-FITC nor PI and shown in the lower left quadrant in flow grams in Figure 2. Each sample was normalized to cells incubated without drug (Medium). Abbreviations: PBMC: Peripheral Blood Mononuclear Cell, FITC: Fluorescein Isothiocyanate, PI: Propidium Iodide.

## Results

*Dose and time-dependent Umbelliprenin induced apoptosis in CLL cells*

**Figure 4:**
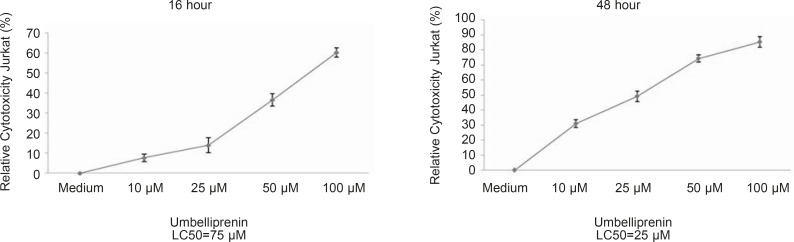
Relative percent cyototoxicity of umbelliprenin on jurkat cells. Umbelliprenin induces apoptosis in a dose- and time-dependent manner (LC_50_, 16 h= 75 µM, LC_50_, 48 h= 25 µM). Data is shown as mean ± standard deviation. Error bars represent 95% confidence interval. Cytotoxicity is defined as: 100-relative viability. Relative viability have been explained in Figure 3. Abbreviations: LC_50_: Lethal Concentration 50%.

I order to determine whether umbelliprenin induces apoptosis in CLL cells, cells were incubated with various concentrations of umbelliprenin *in-vitro* for 16, 20, 24 and 48 h and assayed for apoptosis with annexin V–FITC/PI double staining flowcytometry (Figure 2 and Table1). Staurosporine (1 µM) and the solvent (DMSO, 0.5%) were used as positive and negative controls, respectively. Umbelliprenin induced apoptosis in Jurkat and Raji cells. Interestingly, leukemic cells were more susceptible to this coumarin than normal PBMCs at 10, 25 and 50 µM concentrations of umbelliprenin after 48 h (Figure 3). These findings demonstrate that umbelliprenin induces apoptosis in jurkat T-CLL cells in a dose- and time-dependent manner (LC_50_ varies in different time incubations) (Figure 4) and that raji and jurkat cells are more susceptible to umbelliprenin induced apoptosis compared to normal PBMCs. As Figure 2 and Table 1 show, the highest apoptotic effect of umbelliprenin occurs at 50 µM concentration after 16 h incubation.

**Table 1:** Total apoptosis (Annexin V-FITC positive) induced by umbelliprenin in jurkat cells after 16 and 48 h incubation. Concentration 0 µM means cells incubated without umbelliprenin. Data is shown as mean ± standard deviation.

	**Umbelliprenin concentration (** **µM** **)**	**0**	**10**	**25**	**50**
Time (hour)	16	5.1±1.37	12.20±2.22	13.01±2.82	36.78±1.66
	48	4.59±1.41	24±1.41	23.08±1.56	9.31±0.66

*Apoptotic effect of Umbelliprenin in the presence of IL-4*

**Figure 5:**
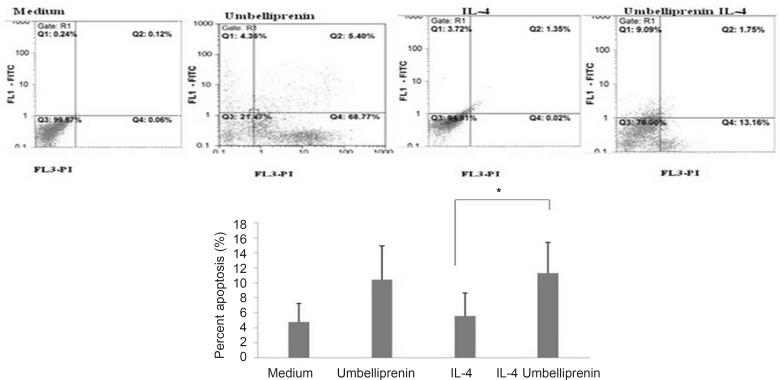
Umbelliprenin (50 µM) retains its apoptotic activity in the presence of IL-4 after 48 h incubation. Data is shown as mean ± standard deviation. *p < 0.05 when compared IL-4 group with IL-4+ umbelliprenin group. Abbreviations: IL-4: Interleukin-4.

To determine whether umbelliprenin could induce apoptosis in Jurkat cells in the presence of IL-4, Jurkat cells were pretreated with IL-4 for 24 h, after which umbelliprenin was added. 48 h later, cells were analyzed for apoptosis. Umbelliprenin keeps its apoptotic effect in the presence of IL-4 (Figure 5). As shown, after 48 h of incubation with umbelliprenin in the presence of IL-4, viability decreases from 94.91% to 76%, and the percentage of apoptosis increases from 5.07% to 10.84%. These data indicate that umbelliprenin remained active in the presence of IL-4. Thus, IL-4 does not increase the *in-vitro* drug resistance of CLL cells exposed to umbelliprenin, in contrast to other drugs that induce apoptosis of CLL cells, such as fludarabine.

## Discussion

Umbelliprenin is produced in various Ferula species (4-6). It has also been found in various plant species consumed as food or used for food preparation (*e.g.* celery). Ferula species used include, *Angelica archangelica*, *Coriandrum sativum* and *Citrus lemon* (13). Natural products have been the source of many medically beneficial drugs, and their importance in the prevention and treatment of cancer is becoming increasingly apparent. Natural products, including bryostatin 1, triterpenoids, and epigallocatechin gallate (EGCG), a polyphenol found in green tea, and their synthetic derivatives have previously been reported to demonstrate activity against B-CLL cells, and some of these compounds have entered clinical trials for treatment of B-CLL and other indolent B-cell malignancies (14-17). In addition, a Chinese herbal extract associated with a sustained complete remission in a B-CLL patient showed direct cytotoxicity to B-CLL cells in vitro (18). Thus, there is reason to consider the use of medicinal botanicals and other natural compounds, perhaps in combination with existing therapies, in the treatment of CLL.

The cytotoxicity of umbelliprenin has been found to be related to the presence of the aliphatic sesquiterpenoid group linked at C7-OH. However, additional *in-vitro* tests and, assays in animal model, are needed to determine the toxicity and the effectiveness of umbelliprenin. In a recent study, Barthomeuf *et al.* studied the induction of apoptosis in a number of human carcinoma lines: colon (DLD1), breast (MCF7), ovary (PA1), prostate (PC3) and non-small cell lung (A549) carcinoma, on primary human fibroblasts and on human metastatic pigmented malignant melanoma M4Beu cells. They found that the level of inhibition varied with the cell line. The cell susceptibility to umbelliprenin decreased in the order: M4Beu>A549=PC3>PA1>fibroblasts= MCF7>DLD1. The IC_50_ of umbellprenin in M4Beu cells after 48 h incubation was 12.4 µM. We found that the IC_50_ of umbelliprenin in Jurkat cells after 48 h incubation was 25 µM. Moreover, they found the number of early apoptotic cells as 17.7% in M4Beu cultures instead of 1.9% in fibroblast, after 48 h incubation with 25 µM concentration of umbelliprenin (7). We also found the number of early apoptotic cells as 19.4% in Jurkat cultures, instead of 0% in PBMCs, after 48 h incubation by 25 µM concentration of umbelliprenin. 

CLL is a disease characterized by the accumulation of apoptotic resistant lymphocytes. The natural product umbelliprenin has been reported to induce apoptosis in a variety of tumor cells. Therefore, we investigated whether umbelliprenin could overcome the apoptotic resistance inherent in CLL cells. We found that umbelliprenin acts directly on CLL cells to induce cytotoxicity in a manner that causes apoptosis within different times. Our data, the first in Jurkat and Raji cells, support previous work demonstrating the activity of umbelliprenin in cell lines and tumor models (7). Umbelliprenin induced cytotoxicity toward Jurkat and Raji cells at concentrations that are minimally toxic to normal PBMCs.

 We showed that incubation of CLL cells with IL-4 couldn`t inhibit the apoptotic effect of umbelliprenin. This phenomenon should be considered in future studies on apoptotic effects of umbelliprenin in the treatment of CLL.

Umbelliprenin may be proposed as an effective therapeutic agent in the treatment of CLL, and thus its application in clinical studies may be considered. Given its ability to overcome apoptotic resistance, umbelliprenin may also be effective in other hematopoietic malignancies. Further investigation of umbelliprenin in mouse models of CLL and other leukemias will contribute to additional understanding of its *in-vivo* activity toward malignant cells and its potential toxicity toward normal tissues. Finally, analysis of the mechanism of action of umbelliprenin in apoptosis pathways and elucidation of its molecular target are greatly needed and they are the subject of our presently ongoing research.
